# Severe community-acquired adenovirus pneumonia treated with oral ribavirin: a case report

**DOI:** 10.1186/s13104-016-2370-2

**Published:** 2017-01-18

**Authors:** Byung Woo Yoon, Yong Geon Song, Seung Hyeun Lee

**Affiliations:** 1 Department of Internal Medicine, Hanil General Hospital, Seoul, Republic of Korea; 2 Department of Internal Medicine, Kyung Hee University School of Medicine, Seoul, Republic of Korea; 3 Department of Pulmonary and Critical Care Medicine, Kyung Hee University School of Medicine, Kyungheedae-ro 23, Dongdaemun-gu, Seoul, 02447 Republic of Korea

**Keywords:** Adenovirus, Ribavirin, Pneumonia, Case report

## Abstract

**Background:**

Adenovirus is a common pathogen of acute upper respiratory infection in children and is generally self-limiting. Severe adenovirus infections have been reported in immunocompromised hosts especially bone marrow transplantation recipients due to hematologic malignancy. Severe adenovirus pneumonia in immunocompetent hosts has rarely been reported and optimal treatment has not been established. We report a case of community-acquired severe adenovirus pneumonia which was successfully treated with early administration of oral ribavirin.

**Case presentation:**

A 39 year-old, previously healthy Korean male was admitted with symptoms of cough, myalgia, febrile sensation. Laboratory findings revealed that he had hypoxemia, thrombocytopenia and elevated transaminase. Chest imaging showed a consolidation with pleural effusion, which was rapidly progressed. All microbiological tests were negative except multiplex real-time reverse transcriptase polymerase chain reaction using respiratory specimen, which was positive for human adenovirus. Under the diagnosis of severe adenovirus pneumonia, we started oral ribavirin, which results in complete recovery without any complications.

**Conclusions:**

This case demonstrates that oral ribavirin, instead of other expensive antiviral treatment, could be a good therapeutic option for the severe adenovirus pneumonia at least occurred in immunocompetent hosts.

## Background

Adenoviruses are double-stranded DNA viruses belonging to the family *Adenoviridae.* They cause infections involving the upper and lower respiratory tract, gastrointestinal tract, and conjunctiva [1]. More than 80% of adenovirus infections occur in children under 4 years old because of a lack of humoral immunity [1]. Although adenovirus infections are generally self-limiting, severe and disseminated infections can occur in patients with impaired immunity including organ transplantation recipients, those with human immunodeficiency virus, and those with congenital immunodeficiency. Outbreaks of adenovirus pneumonia in immunocompetent patients have occasionally been reported among military recruits and adults in long-term care facilities or hospital wards, which included some fatal cases [2–4]. However, community-acquired adenovirus pneumonia in immunocompetent adults has rarely been described. Herein, we report a case of severe adenovirus pneumonia occurring in a previously healthy man which was successfully treated with oral ribavirin.

## Case presentation

**Fig. 1:**
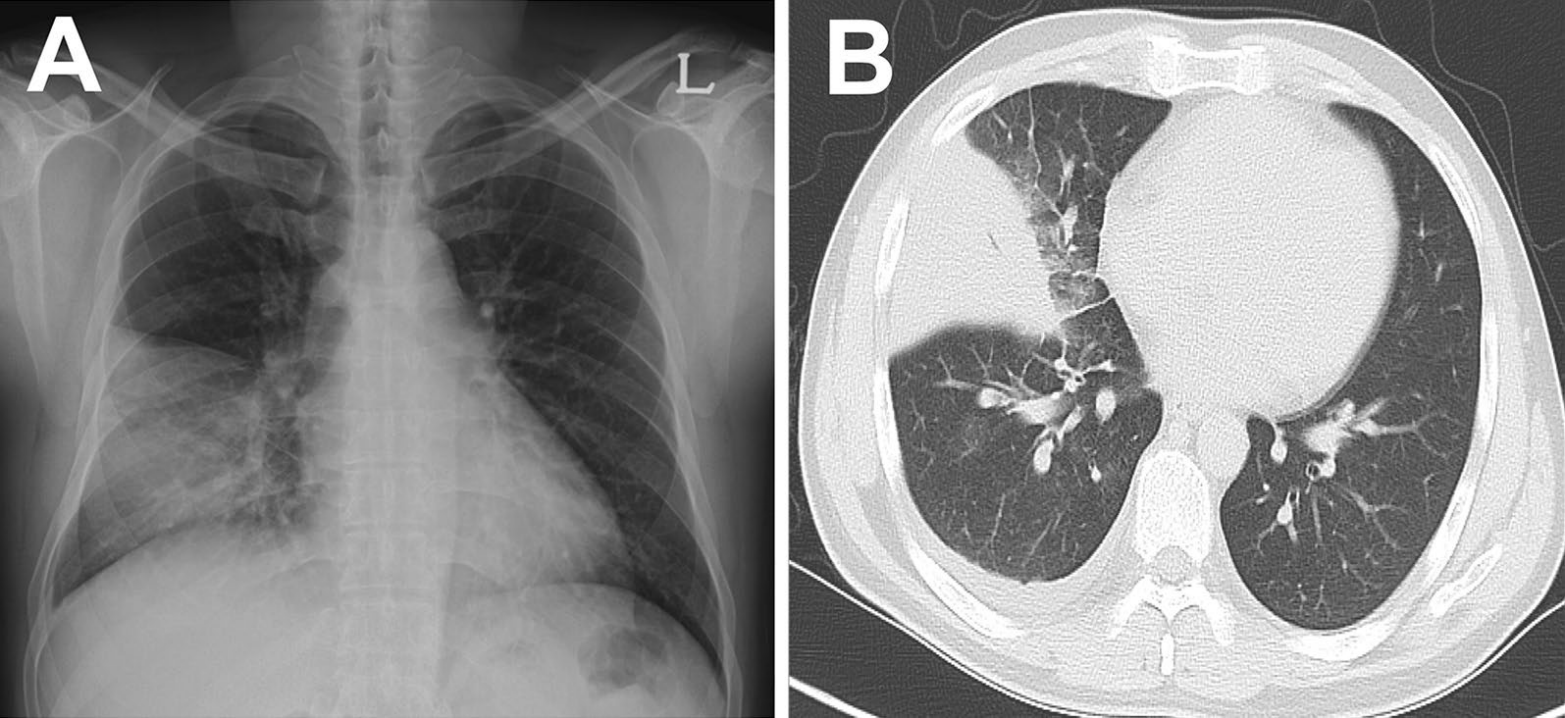
Chest images at presentation. Chest X-ray showed a consolidation in the right mid to lower lung fields (**A**). Chest computed tomographic scan showed dense consolidation with surrounding ground glass opacities in the lateral segment of the right middle lobe and a small amount of pleural effusion in the right hemithorax (**B**)

**Fig. 2:**
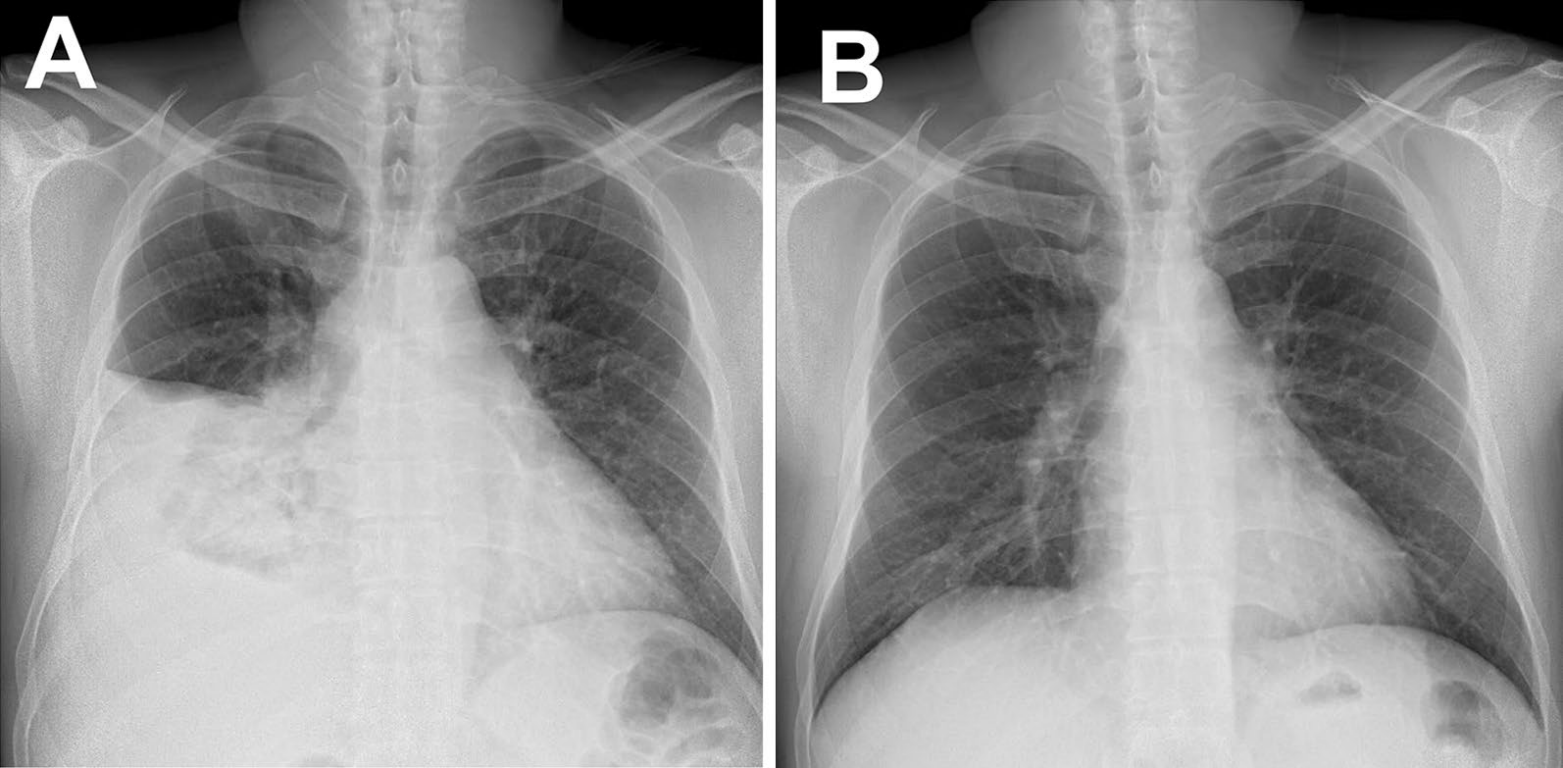
Follow-up chest X-rays. A chest X-ray on hospital day 2 showed rapid progression of the consolidation and pleural effusion (**A**). After administration of ribavirin, the lesions were completely resolved on hospital day 13 (**B**)

**Fig. 3:**
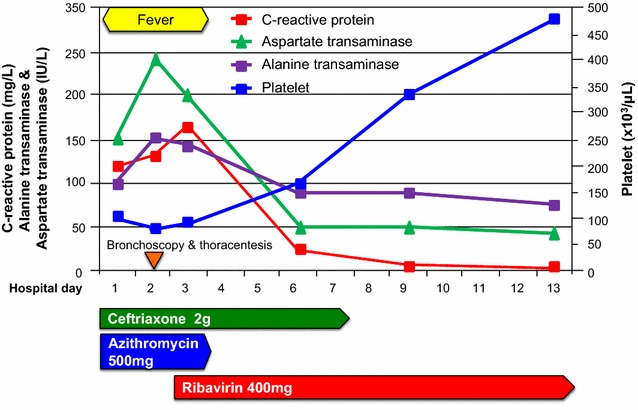
Summary of clinical course. After administration of ribavirin, the fever subsided and laboratory abnormalities gradually improved

A 39-year-old Korean male come to our hospital complaining of cough, myalgia, and fever that had lasted for 5 days. He was a company worker and denied any previous medical histories. He was a current smoker and drank alcohol about once a month. His vital signs were: blood pressure, 100/60 mmHg, heart rate, 100/min, respiratory rate, 25 breaths/min, and body temperature, 39 °C. On the physical examination, decreased breathing sound was noted in the right lower lung. Laboratory tests revealed a c-reactive protein (CRP) level of 119 mg/dL, a total bilirubin level of 1.8 mg/dL, and alanine transaminase and aspartate transaminase levels of 250 and 172 IU/L, respectively. His platelet count was 98,000/mm^3^, while his white cell count was 8150/mm^3^ (neutrophil: 85%). In the arterial blood gas analysis checked in room air, pH, PaCO_2_, PaO_2_, bicarbonate, and oxygen saturation levels were 7.50, 34 mmHg, 67 mmHg, 26.5 mmol/L, and 95%, respectively. A test for the human immunodeficiency virus was negative. Mycoplasma and Chlamydia antibodies were negative. Streptococcal and Legionella urinary antigens were negative. Anti-nuclear and anti-neutrophilic cytoplasmic antibodies were also negative. A chest X-ray showed consolidation in the right mid to lower lung fields. Chest computed tomography showed consolidation with surrounding ground glass opacity in the right middle lobe with a small amount of pleural effusion in the right hemithorax (Fig. 1). Abdominal sonography revealed no abnormal finding in the hepatobiliary system. We began to administer 4 L/min of oxygen nasally and empirical antibiotics with third generation cephalosporin and macrolide following a diagnosis of community-acquired pneumonia. On the second day in the hospital, the patient’s fever was sustained and he complained of dyspnea. His hypoxemia was aggravated such that he required 7 L/min of oxygen via a simple mask and the consolidation and pleural effusion had markedly progressed (Fig. 2a). We performed bronchoscopy and thoracentesis. Multiplex real-time reverse transcriptase polymerase chain reaction (RT-PCR) for respiratory viruses using bronchoalveolar lavage fluid was positive for human adenovirus while other microbiological studies were negative. Pleural fluid was lymphocyte-dominant exudate and was also positive for human adenovirus. Under the diagnosis of adenovirus pneumonia, we started antiviral therapy with oral ribavirin 400 mg q 12 h while maintaining antibiotics. On hospital day 4, his fever had subsided and symptoms were much improved. The transaminase levels, CRP and platelet counts gradually normalized (Fig. 3). A follow-up chest X-ray was clear (Fig. 2b) and he was discharged in hospital day 13 without any complications.

## Discussion and conclusions

Adenovirus causes 1–7% of adult respiratory tract infections and present as non-specific febrile illness which subsides spontaneously within several days [1]. Adenovirus pneumonia occasionally occurs in immunocompromised hosts and is characterized by rapid clinical deterioration compared to other viral pneumonias with a reported fatality rate of 50%. Community-acquired adenovirus pneumonia in immunocompetent hosts is very rare.

A recent review of 21 cases of community-acquired adenovirus pneumonia revealed that patients frequently have respiratory compromise with hypoxemia at the time of presentation, while the classical features of adenoviral infection such as pharyngitis, conjunctivitis, rash, or diarrhea, are usually absent [5]. Most patients deteriorate rapidly after admission and require intubation and mechanical ventilation. Laboratory findings are typical of viral infections; a normal white blood cell count, relative lymphopenia, thrombocytopenia and elevated transaminase are frequently observed [5]. Although widespread bilateral interstitial shadowing was the most common radiologic finding, lobar consolidation was observed in about one quarter of the cases [5].

Traditionally, adenovirus infections are diagnosed using viral cultures and virus-specific immunofluorescent stains, which are time-consuming and costly. The recently-adopted multiplex real-time RT-PCR assay is beneficial for its rapid and highly sensitive detection of different respiratory pathogens simultaneously. In this case, initially atypical presentation and rapidly progressing clinical manifestations led us to suspect atypical pneumonia including viral pneumonia and we performed the assay, which allowed for very early detection of the pathogen and initiation of antiviral therapy, which was critical for the favorable clinical outcome.

The optimal treatment for severe adenovirus infections has not been established. A recent study reported that cidofovir, a nucleotide analogue, showed possible effect in some immunocompetent patients [6]. However, it is expensive and is not easily accessible in our country, and severe side effects including renal and hematologic toxicity should be considered. Ribavirin, a nucleoside analogue, is less toxic and has long been used for the treatment of hepatitis C. Although it has shown efficacy in treating severe adenovirus infections in infants, children, and immunocompromised adults, only high dose intravenous ribavirin was used in those cases [1, 4, 7, 8]. Despite of thorough review of literatures, the clinical efficacy of oral ribavirin for the community-acquired adenovirus pneumonia has not been reported. Although two case series reported that ribavirin was used in some immunocompetent patients for severe community-acquired adenovirus pneumonia, the treatment outcome according to the use of ribavirin and the administration route of ribavirin are not described in either of those studies [9, 10]. Our case clearly describes the successful use of oral ribavirin in the treatment of community-acquired adenovirus pneumonia in a previously healthy patient. Although more studies are required to determine its efficacy, oral ribavirin could be considered as a therapeutic option especially in the cases where cidofovir or intravenous ribavirin is not readily available.

In conclusion, community-acquired adenovirus pneumonia is a rare but rapidly deteriorating condition. Clinical suspicion and early detection using multiplex real-time RT-PCR is critical for the diagnosis and rapid initiation of treatment. Our case suggests that oral ribavirin could be a therapeutic option for adenovirus pneumonia at least in immunocompetent patients, and facilitates future randomized trial to prove its efficacy in those population.

## Abbreviations

CRP: c-Reactive protein
RT-PCR: reverse transcriptase polymerase chain reaction
